# Factors Influencing Implementation of the Commission on Cancer’s Breast Synoptic Operative Report (Alliance A20_Pilot9)

**DOI:** 10.1245/s10434-024-15515-2

**Published:** 2024-06-11

**Authors:** Ko Un Park, Tasleem J. Padamsee, Sarah A. Birken, Sandy Lee, Kaleigh Niles, Sarah L. Blair, Valerie Grignol, Diana Dickson-Witmer, Kerri Nowell, Heather Neuman, Tari King, Elizabeth Mittendorf, Electra D. Paskett, Mary Brindle

**Affiliations:** 1 Division of Breast Surgery, Department of Surgery, Brigham and Women’s Hospital, Boston, MA USA; 2 Ariadne Labs, Brigham and Women’s Hospital, Harvard T.H. Chan School of Public Health, Boston, MA USA; 3 Breast Oncology Program, Dana-Farber/ Brigham Cancer Center, Boston, MA USA; 4 Harvard Medical School, Boston, MA USA; 5 James Comprehensive Cancer Center, The Ohio State University, Columbus, OH USA; 6 Wake Forest University School of Medicine, Winston-Salem, NC USA; 7 University of California San Diego, La Jolla, CA USA; 8 Beebe Healthcare, Rehoboth Beach, DE USA; 9 Physicians’ Clinic of Iowa, Cedar Rapids, IA USA; 10 School of Medicine and Public Health, University of Wisconsin, Madison, WI USA; 11 Department of Surgery, Alberta Children’s Hospital, Cumming School of Medicine, University of Calgary, Calgary, Canada

## Abstract

**Background:**

The technical aspects of cancer surgery have a significant impact on patient outcomes. To monitor surgical quality, in 2020, the Commission on Cancer (CoC) revised its accreditation standards for cancer surgery and introduced the synoptic operative reports (SORs). The standardization of SORs holds promise, but successful implementation requires strategies to address key implementation barriers. This study aimed to identify the barriers and facilitators to implementing breast SOR within diverse CoC-accredited programs.

**Methods:**

In-depth semi-structured interviews were conducted with 31 health care professionals across diverse CoC-accredited sites. The study used two comprehensive implementation frameworks to guide data collection and analysis.

**Results:**

Successful SOR implementation was impeded by disrupted workflows, surgeon resistance to change, low prioritization of resources, and poor flow of information despite CoC’s positive reputation. Participants often lacked understanding of the requirements and timeline for breast SOR and were heavily influenced by prior experiences with templates and SOR champion relationships. The perceived lack of monetary benefits (to obtaining CoC accreditation) together with the significant information technology (IT) resource requirements tempered some of the enthusiasm. Additionally, resource constraints and the redirection of personnel during the COVID-19 pandemic were noted as hurdles.

**Conclusions:**

Surgeon behavior and workflow change, IT and personnel resources, and communication and networking strategies influenced SOR implementation. During early implementation and the implementation planning phase, the primary focus was on achieving buy-in and initiating successful roll-out rather than effective use or sustainment. These findings have implications for enhancing standardization of surgical cancer care and guidance of future strategies to optimize implementation of CoC accreditation standards.

**Supplementary Information:**

The online version contains supplementary material available at 10.1245/s10434-024-15515-2.

The outcomes for patients who undergo cancer surgery are associated with the technical aspects of this operation (i.e., completeness of resection and margins). Standardizing operative technique can lead to improved outcomes for patients with cancer.^1,2^ The Commission on Cancer (CoC) is an accreditation program that includes 1500 accredited cancer centers that treat 70 % of Americans with cancer. In 2020, the CoC accreditation standard underwent major revisions to include the performance and documentation of key elements of cancer surgery, including axillary surgery for patients with breast cancer.^3^

As part of the new accreditation standard, the CoC requires that key elements be documented in a checklist-type electronic synoptic operative report (SOR) to facilitate education, integration, documentation, and monitoring of adherence to the surgical standards. Breast SOR includes specific technical elements for sentinel lymph node biopsy (SLNB) and axillary lymph node dissection (ALND).^4,5^

The expectation from the CoC was that the programs would develop an implementation plan by 2022. As of January 2023, surgeons are required to include an additional SLNB or ALND SOR into their operative report if these surgeries were performed in order for institutions to maintain CoC accreditation.^4^ To meet the requirements, the SOR must list the required data elements in a specific format. Responses in a prose format do not meet the requirements.

Synoptic reporting is a way of standardizing and improving cancer care.^6–9^ The use of a checklist-based data collection form in synoptic reporting has clinical benefits. Improved efficiency and complete documentation of key elements facilitate the finding of important information by other cancer and non-cancer providers. Easier abstraction of information also is important for quality measurements and research.^6–8,10,11^

The new SOR standard has the potential to play a substantial role in improving the quality of care for patients with breast cancer. To realize the potential benefits, CoC programs need to successfully implement the standard. Past CoC standards such as psychosocial distress screening, survivorship care plans, and tobacco cessation plans have failed to demonstrate large-scale improvements in patient outcomes despite strong evidence supporting their value.^5,12–16^ In part, the limited benefit can be attributed to uneven success of implementation within CoC-accredited cancer programs.

Based on these examples, we believe successful integration of breast SOR hinges on implementation strategies designed to target implementation barriers.^5^ As a first step in this qualitative study, we interviewed key informants from a variety of CoC programs to gain an understanding of the barriers and facilitators to implementation of the breast surgical standards.

## Methods

From December 2021 to May 2022, we conducted in-depth semi-structured interviews with health care professionals involved in SOR implementation to investigate the factors influencing implementation of breast SOR.

### Site Selection

We used purposive sampling to identify four CoC-accredited institutions that represented varied institution types, electronic medical record (EMR) vendors, and geographic regions of the country. Two sites were Comprehensive Community Cancer Programs (largest CoC accreditation category), which implies the sites have 500 or more new cancer cases each year, and two sites were NCI-Designated Cancer Programs to evaluate the hypothesis that these “high-resource” programs also will experience barriers to implementing the new surgical standards. We approached sites by email, selecting sites interested in participating and sites that had a champion willing to engage in the study.

### Interview Participant Selection

Within participating sites, we recruited key informants involved in breast SOR implementation including breast surgeons, cancer liaison physicians (CLP), cancer program administrators (including oncology data specialists), and information technology (IT) personnel from the health system. A CLP is a physician of any specialty and an appointed person within each CoC program to fulfil the role of the physician quality leader. We also used snowball sampling, asking interviewees to identify additional informants with relevant perspectives on SOR implementation at each site. Participants consented to participate in the study.

### Data Collection

Two researchers (K.P. and S.L.) conducted the virtual interviews (Zoom Video Communications, Inc. San Jose, CA, USA). To develop the interview guide, we used two widely used implementation science determinant frameworks: the Consolidated Framework for Implementation Research (CFIR) and the Theoretical Domain Framework (TDF).^17–19^ The combined use of CFIR-TDF guided the assessment of implementation barriers and facilitators at the individual, organizational, and external levels, including the role of CoC.^20^ The interviews were audio-recorded and transcribed verbatim using NVivo transcription (Lumivero, Denver, CO, USA). We de-identified transcripts for analysis.

### Data Analysis

Two qualitative analysts (S.L. and K.N.), with guidance (K.P. and T.J.P.), coded and analyzed the data using NVivo (version 12). Deductive coding and template analysis were used based on a priori themes from CFIR and TDF domains as our initial codebook (Supplemental file).^5,20^ The two coders (S.L. and K.N.) coded a portion of the transcript independently, convened to discuss and clarify the codes, and resolved the discrepancies (with K.P.). If codes were found that did not fit into the a priori themes from CFIR and TDF, additional codes were allowed to emerge until the codebook was finalized.

To measure the agreement between the coders, Cohen’s kappa coefficient was queried after the initial coding. Negative Cohen’s kappa values were reviewed, and discrepancies were resolved through discussion until the Cohen’s kappa values were greater than zero. Then, S.L. and K.N. identified themes within each code. Disagreements on thematic synthesis were resolved through team discussion and review of the transcript and coded data. The themes were further consolidated into four main categories.

Data were reported according to the Standards for Reporting Qualitative Research (SRQR) guidelines.^21^ This study was approved by The Ohio State University institutional review board, and the study was conducted in accordance with the Declaration of Helsinki.

## Results

We conducted 31 interviews with key informants from four CoC programs (Table 1). The interviews include 10 surgeons, 4 CLPs, 11 cancer program administrators, and 6 IT personnel. Three of the sites (institutions B, C, and D) used Epic as the singular EMR system, and one site (institution A) had a combination of different EMR systems including Cerner.

**Table 1:** Characteristics of participating CoC-accredited institutions

Institution characteristic	Participating CoC-accredited institutions			
	Institution A	Institution B	Institution C	Institution D
Cancer program category	Comprehensive community cancer program	NCI designated comprehensive cancer center	NCI designated comprehensive cancer center	Comprehensive community cancer program
Location	East	Midwest	West	Midwest
Organizational control	Non-profit	Non-profit	Non-profit	Non-profit
EMR type	Athena, Cerner	Epic	Epic	Epic
Employed breast surgeons (*n*)	2	8	3	0
Independent surgeons (*n*)^a^	0	0	0	5

CoC, Commission on Cancer; NCI, National Cancer Institute; EMR, electronic medical record

^a^Breast surgeon not employed by cancer program

Despite CoC’s expectation that programs would have developed an implementation plan by 2022, during the period of the interviews (December 2021 to May 2022), none of the sites had implemented breast SOR, and no surgeons had previously used breast SORs. The CoC program leaders were aware of the breast SOR accreditation requirement but had not made any explicit implementation decisions.

Informants reported barriers to implementing breast SOR across all CFIR domains and several TDF constructs. Representative quotes are outlined in the Supplemental file. Two additional themes emerged: one a facilitator (non-breast SOR) and one a barrier (uncertainty surrounding the accreditation requirements). First, informants referred to their “non-breast SOR” experience with implementing colorectal accreditation standards and creating a registry as a facilitator of the implementation of breast SOR. This was considered an attribute different from “knowledge,” “self-efficacy,” or “belief about capabilities” themes in TDF. Surgeon informants described their reading of synoptic pathology reports as another “non-breast SOR”-facilitating experience.

Second, in describing a barrier, the CoC program leaders expressed uncertainty surrounding the exact requirements for the breast SOR and the timeline for meeting them (because the CoC was continuing to change them) and thought that the CoC had not provided sufficient information about implementation and monitoring compliance.

One informant said:

“So initially it was for sure lack of direction, and I think confusion because the Commission on Cancer kept talking about like an interface for an API with our electronic medical records, but never really talked through what that was and what that looked like. So that in my mind, it was like, what does that mean?”

**Fig. 1:**
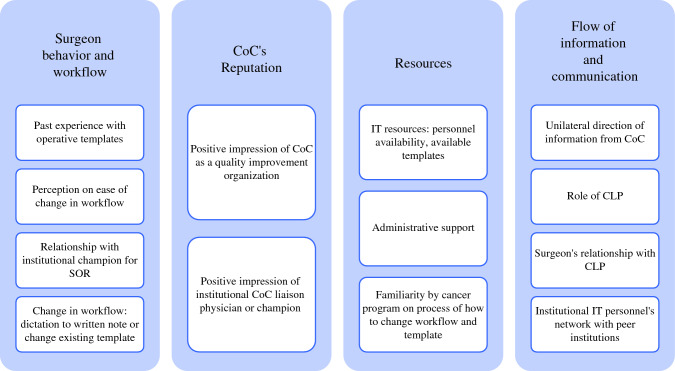
Examples of specific factors influencing implementation of breast SOR. CLP, cancer liaison physician; CoC, Commission on Cancer; SOR, synoptic operative report

**Fig. 2:**
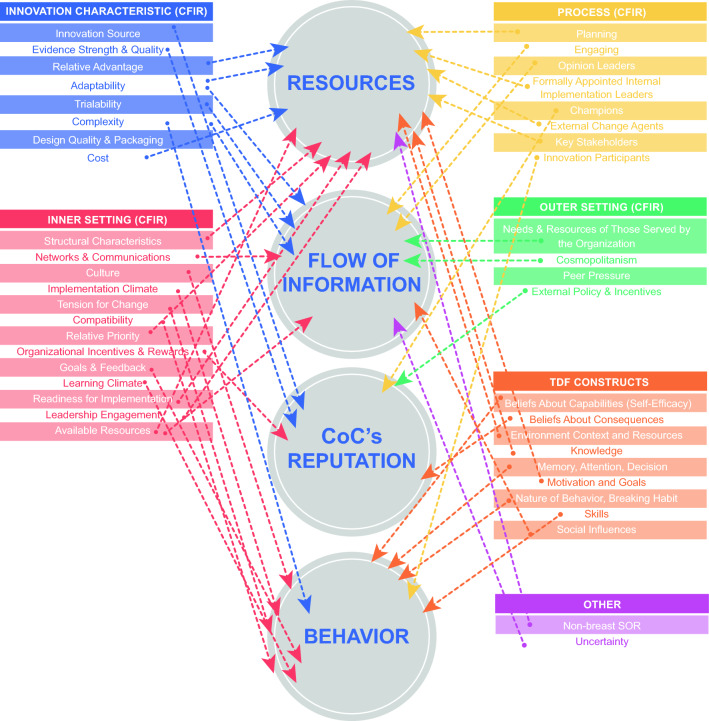
CFIR and TDF codes influencing implementation of a breast synoptic operative report mapped into four major categories. CFIR, Consolidated Framework for Implementation Research; TDF, Theoretical Domain Framework

We identified the following four key overarching categories of factors influencing breast SOR implementation: behavior, the CoC’s reputation, resources, and flow of information (Figs. 1 and 2).

### Surgeon Behavior and Workflow Integration Influenced Implementation Success

Surgeon behavior, workflow change, and willingness to change were barriers discussed by informants from all four participating institutions. Workflow changes involved switching from prose-type operative notes to a templated checklist note that incorporated the synoptic element. Key informants discussed multiple factors that influence change in behavior, including past experience with templates, perception of how easy it would be to change the existing workflow, and relationships with the person championing the breast SOR initiative at his or her institution. The following representative quotes reflect these factors:

“I think that our EHR [electronic health record] systems and our ability to do them from a logistics standpoint is not—is not going to fit in immediately with our workflow. And so changing habits, especially, I think for surgeons is very difficult. For anybody it’s difficult, but I don’t know; I think surgeons are especially stubborn.”

“We have talked about templating our notes in the past, and the reception for that was mixed because a lot of people have their notes in such a way that it works with their workflow, and they believe it’s more efficient.”

“. . . All my notes are pre-templated and then I alter components of it that are different for each case. . . . I would just update that language to reflect the CoC's requirements. So, it would require me sitting there for maybe 30 minutes and updating my four templates.”

“I guess at the institutional level through our cancer committee, just the people that I interact with a lot, a couple of them have been the ones who have reached out to me about [the SOR]. So, I would say . . . that’s had a positive influence because it's people that I'm working with all the time.”

### CoC’s Reputation Enhanced Buy-in

The CoC’s positive reputation circumvented a perceived lack of evidence supporting breast SOR and reinforced the need to implement SOR. Informants mentioned the potential for improving data collection and research in general. When asked about the evidence supporting the breast SOR, many discussed not knowing the explicit evidence on SOR improving quality of care:

“I understand the value of discrete documentation and all of the downstream effects that make that beneficial. But in terms of clinical care, I can only assume. I can’t say that I’ve read, you know, because I don’t know that it’s implemented across the board to—to have evidence.”

Despite not knowing the evidence to support breast SOR, informants discussed having positive impressions of the SOR because of the CoC’s reputation as an accreditation body that supports quality improvement:

“They all know that, you know, like the recommendations are typically evidence based. So, there is respect that if the CoC is requiring certain things that there’s good cause for it, you know, so I think it actually ends up legitimizing some of the things that we want to change.”

### Prioritization of Resources Was Necessary for Timely Adoption

Resource-related requirements included not only having IT personnel with relevant experience, but also IT prioritization of the breast SOR initiative. Informants noted that the perspectives of institutional leaders did not always align with CoC initiatives because there were no explicit immediate monetary benefits to the organization obtaining CoC accreditation. Without explicit prioritization at the higher level, key informants noted needing to wait for the template to be built into the EMR because IT personnel are a shared resource throughout the entire institution:

“You know, it was kind of a struggle . . . with the C-suite because [implementing the SOR] wasn’t identified as something that was very important, and it wasn’t clear to the C-suite that there was going to be a return on investment. . . . So, it’s understanding that the institution has a very concrete concept of return on investment, and it’s typically monetary. As physicians, I feel like we would do anything that’s the best for the patient. . . . We don't think about how much it costs. But the C-suite administrators think about the cost.”

Additional personnel and time resource constraints were noted that were related to the COVID-19 pandemic and the necessity for hospitals to respond to the pandemic by redeploying personnel to tasks other than those to which they would normally be assigned. Given the competing demands for personnel and time resources due to the COVID-19 pandemic, some criticisms focused on the timing of the CoC’s launch of the new standards:

“So, in the middle of that surge, how do you juggle, especially if you’re in a place that's canceling surgeries because of some of the things going on with COVID; how do you manage some of these educational things and making sure that they’re being done appropriately when half of your staff could be out based on so many things going on in the community? . . . It's just . . . a very interesting time to really be rolling out anything new on health care right now, especially something that may not have—would have been just as beneficial if you would have waited a little bit longer.”

### Reliable, Multidirectional Information Flow Was Needed to Support Implementation

The unidirectional flow of information about SOR from the CoC, mainly through emails to the CLPs and communication with peer networks, influenced implementation. The CoC’s major announcements typically were distributed first to the CLPs, but the surgeons’ external networks also contributed as a source of information. It was the responsibility of the CLPs not only to distribute the information, but also to help design the implementation plan within their institutions. One cancer program administrator noted:

“. . . The biggest is email communication [from the CoC]. We also get communication from our liaison physician; that’s Dr. [name of CLP].”

Although webinars also were published by the CoC, one institution noted that some of the information was available only to those with CoC login access, which made it challenging to share the information with necessary institutional personnel involved with the implementation (e.g., IT personnel):

“So that little brief video I found helpful to basically level set everybody: our chairman, all the physicians that participate, as well as the other members of Cancer Committee. So, we used that initial YouTube video that was, I believe I found that link on the CoC website, to just educate everybody as to what the expectation is for the next three years, roughly. And then from that, Dr. [name of CLP] and I met with our technology team, our IT team, and he had access that through data links that we shared our screen and showed them the whole video.”

Informants felt they experienced great benefit networking with peer institutions who had already successfully implemented the breast SOR. According to IT personnel, opportunities were available through EMR vendors such as Epic and Cerner to be able to learn from peer institutions:

“. . . If we can see what other folks have done, you know, we don’t have to recreate the wheel. . . . I think in our meetings with departments, one of the most common questions I hear is, well, what are other hospitals doing? . . . We hear that pretty frequently.”

## Discussion

This study provides an in-depth exploration of factors that influence implementation of the new breast SOR accreditation standard. Informants provided rich insights into the multifaceted challenges associated with the implementation of SOR. Our examination of the findings underscored the critical role of anticipating surgeon resistance to workflow changes, capitalizing on CoC's reputation, anticipating resource needs, and facilitating the flow of information to support the successful integration of SOR.

A substantial body of literature underscores the significance of standardized reporting as an important element in efforts to improve cancer care. In a U.S. study on surgery for rectal cancer, use of SOR-educated surgeons for the important elements in formal cancer resection ensured that the necessary steps of a sound cancer operation occurred by acting as a checklist of reminders.^22^

Our study's findings align with previous research on CoC standard implementation that highlights the pivotal role of behavior change and the need for dedicated resources to implement new practices.^22–24^ A recent qualitative study of general surgeons in Iowa showed that they were unfamiliar with the CoC standards and expressed skepticism about the importance of the new surgical standards.^25^ Surgeons expressed concern about the organizational burden of maintaining CoC accreditation. The multifaceted nature of implementation challenges, spanning individual attitudes, institutional priorities, resource allocation, and communication strategies, is consistent with literature on implementing other complex interventions.

Within health care, there is a tendency to focus on “education” when there are new initiatives underway.^26^ However, the recognition of behavior, CoC’s reputation, resources, and the flow of information as core determinants highlights that multi-dimensional strategies are needed to address implementation challenges.

One of the main elements missing in the implementation discussion from all four institutions in this study, perhaps because of a focus on initial adoption and buy-in, was an explicit process for monitoring compliance with SOR use. Despite the great emphasis placed on designing and introducing the SOR into the surgeon’s operative report, a concurrent audit-feedback and monitoring plan was not discussed as an explicit part of the organizations’ initial implementation plan (even though programs need to demonstrate 80 % compliance to meet accreditation standards). It was evident that during the early implementation and the implementation-planning phase, the programs were focused primarily on achieving buy-in and initiating successful roll-out rather than on integration of workflow with an auditing mechanism and its effective use. A Cochrane review showed the benefit of audit feedback in increasing adoption of target behaviors by clinicians, especially when the baseline performance was low. The institution benefits when the source of feedback is a supervisor or colleague, the feedback is delivered more than once, the feedback is delivered in different formats (verbal and written), and the audit-feedback process includes both explicit targets and an action plan.^27^ Future studies evaluating audit feedback as an implementation strategy to augment breast SOR use are currently underway.

Our findings should be interpreted together with several caveats including the nature of social desirability bias in key informants participating in the interview. The findings are from three institutions that use Epic, and the experiences elsewhere could be different. However, given the rigor of the frameworks used for analysis and the range of themes evaluated in this study, we believe the key elements elucidated have wide implications.

This study also focused explicitly on the breast SOR standards and not on other surgical standards such as colorectal, thoracic, or melanoma standards, which were beyond the scope of this study. However, this study reported findings that are generalizable to other surgical standards (e.g., the limitation in IT resources and the CoC’s reputation enhancing buy-in). Because we anticipate that the workflow changes for breast surgery likely differ from those for colorectal cancer or melanoma, in the designing of implementation strategies, these differences should be taken into account.

In conclusion, our study’s comprehensive exploration of factors influencing implementation of breast SOR sheds light on the intricacies of adapting standardized reporting practices in cancer care. The use of CFIR and TDF frameworks allowed for a comprehensive exploration of individual and organizational determinants, offering insights into the implementation process. Key insights gained from this qualitative assessment showed that CoC standards should be paired with explicit guidance for implementation tailored to the unique challenges associated with SOR implementation. This will require implementation research before issuance of new standards.

The findings of this study can be transformed into potential courses of action such as implementation toolkits focused on guidance for changing clinical workflow, anticipating resource requirements, and capitalizing on the CoC’s reputation. Further work evaluating implementation strategies that may help increase the uptake and decrease the burden of implementing the CoC’s surgical standards currently is underway.

### Data-Sharing Statement

Deidentified participant data and qualitative interview coding data will be available upon request sent to the PI (kpark16@bwh.harvard.edu) after publication. The data will be made available to researchers whose proposed use of the data has been approved and after the completion of a signed data access agreement.

### Supplementary Information

Below is the link to the electronic supplementary material.

Supplementary file1 (DOCX 80 kb)
