# Supplementary material for: Factors Influencing Implementation of the Commission on Cancer’s Breast Synoptic Operative Report (Alliance A20_Pilot9)
Source: Ann Surg Oncol. 2024 Jun 11;31(9):5888–95. doi: 10.1245/s10434-024-15515-2 (PMC11300652; doi:10.1245/s10434-024-15515-2)
Supplement: Supplementary file 1 — Supplementary file1 (DOCX 80 kb) [file 10434_2024_15515_MOESM1_ESM.docx]

**SUPPLEMENTARY MATERIAL**

**Factors influencing implementation of Commission on Cancer’s Breast Synoptic Operative Report (Alliance A20_Pilot9)**

Ko Un Park, MD, FACS; Tasleem J. Padamsee, PhD; Sarah A. Birken, PhD; Sandy Lee, BS; Kaleigh Niles, MPH; Sarah L. Blair, MD, FACS; Valerie Grignol, MD, FACS; Diana Dickson-Witmer, MD, FACS; Kerri Nowell, MD, FACS; Heather Neuman, MD, FACS; Tari King, MD, FACS; Elizabeth Mittendorf, MD, PhD, MHCM, FACS; Electra D. Paskett, PhD; Mary Brindle, MD, MPH, FACS

**Codebook**

**Codebook**

**General Guidance on Coding**

1. Coders should read through the entire codebook prior to coding any transcripts.
2. Coders should read through the transcript before coding.
3. Do not apply Parent Codes; these are domain names for grouping Child Code s together.
4. While coders should aim to apply a single code that best fits the meaning of a passage, multiple codes should be applied when a passage meaningfully describes multiple constructs.
5. Refer to all components of the codebook when coding, including the definition, inclusion criteria, exclusion criteria, and examples for double coding.
6. Consider all codes as “tension codes,” meaning they can be used to code passages referring to the positive or negative, or the presence or absence, of a construct.

**CFIR codes**

| 1. **Innovation Characteristics** |  | Examples |
| --- | --- | --- |
| 1. Innovation Source | Definition: Perception of key stakeholders about whether the innovation is externally or internally developed. [**reference to SOR coming from the CoC]**  Inclusion Criteria:   - Include statements about the source of the innovation and the extent to which interviewees view the change as internal to the organization, e.g., an internally developed program, or external to the organization, e.g., a program coming from the outside. Note: May code and rate as "I" for internal or "E" for external. - Include statements about idea of SOR coming from CoC - Include statements about SOR as an innovation coming from CoC - Include statements about SOR as a standard or requirement from the CoC in order to maintain accreditation. May be double coded + External Policy & Incentives.   Exclusion Criteria: Exclude or double code statements related to who participated in the decision process to implement the innovation to [Engaging](http://cfirwiki.net/wiki/index.php?title=Engaging), as an indication of early (or late) engagement. Participation in decision-making is an effective engagement strategy to help people feel ownership of the innovation.   - Exclude statements general statements about CoC accreditation (no specific mention or reference to SOR) | “ I received the standard CoC emails with any updates and probably at first it was the 2020 phase-in standards. Since then, I have watched the--it wasn't synoptic Op—it was the path synoptic reporting webinar, have been kind of keeping up on all of the updates on whether or not reporting templates would be released from the CoC.” |
| 1. Evidence Strength & Quality | Definition: Stakeholders’ perceptions of the quality and validity of evidence supporting the belief that the innovation will have desired outcomes.  Inclusion Criteria: Include statements regarding awareness of evidence and the strength and quality of evidence, as well as the absence of evidence or a desire for different types of evidence, such as pilot results instead of evidence from the literature.  Include statements about awareness of evidence (or lack of) supporting effectiveness of innovation  Include statements about descriptions of evidence (or lack of) supporting effectiveness of innovation  Double coding:  Double code desriptions of evidence to TDF Knowledge  Double code descriptions of use of results from local or regional pilots to [Trialability](http://cfirwiki.net/wiki/index.php?title=Trialability)  Exclusion Criteria: Exclude or double code statements regarding the receipt of evidence as an engagement strategy to [Engaging](http://cfirwiki.net/wiki/index.php?title=Engaging): Key Stakeholders.   - Exclude statements about innovation itself - Exclude statements about advantages and disadvantages of SOR. Code to TDF Relative Advantage instead. | “ No, I don't know that I have. If I have, I haven't read it as such. I would say in general, in my role, I understand the value of discrete documentation and all of the downstream effects that make that beneficial. But in terms of clinical care, I can only assume. I can't say that I've read, you know, because I don't know that it's implemented across the board to—to have evidence. But if there's evidence, I'd be happy to review it and read it.”  “Well, I think if you look at any sort of meta-analysis that looks at synoptic reports, you see that the accuracy and completeness is better. The speed of them is the same or faster. And so, and so many—there's lots of different studies. It's hard to compare them because they don't all have the same metrics, but I think there's a lot of reasons that are better and it's the reason why AJCC and CAP have gone to more standardized synoptic reports. CAP has been doing it to some extent for 20 years, but within the last five years, they're really, really dedicated to the standardized pathology sort of output report. So. Yeah, there's data behind it.”  “Well, I guess in terms of networking, you know, the people who are on the committees and spearheading synoptic reporting are very well-respected clinicians, researchers in their field. So, I think from that standpoint, it lends credence and legitimacy to the adoptance—adoption of the synoptic reporting.” |
| 1. Relative Advantage | Definition: Stakeholders’ perception of the advantage of implementing the innovation versus an alternative solution.  Inclusion Criteria: Include statements that demonstrate the innovation is better **(or worse)** than existing programs.   - Include hypothetical advantage or disadvantage   Exclusion Criteria: Exclude statements that demonstrate a strong need for the innovation and/or that the current situation is untenable and code to [Tension for Change](http://cfirwiki.net/wiki/index.php?title=Tension_for_Change).   - Note updated definition of TDF Knowledge | “Well dictated reports is what we all are used to so it’s fast and you do it. The disadvantages—you can forget things like I personally, this is probably not the right thing to do, but I usually am running from one room to the other and I dictate all my notes at the end of the day. So, I probably forget some details. So, if it was like a check box then at least I’ll have to remember all that stuff. And I’m hoping it will be quicker. I mean I dictate pretty fast actually. Um yeah, I mean it’s easier to check a box than to dictate. I’m hoping.”  “Well, I think the missingness and accuracy are probably the really big things. There's some potential benefit in other areas where we should see that synoptic reporting can be actually more—or more quick to do the non-narrative approach. So those are the big, across synoptic reports, those are the big advantages.” |
| 1. Adaptability | Definition: The degree to which an innovation can be adapted, tailored, refined, or reinvented to meet local needs.   - Code adaptability only for flexibility of innovation itself to meet local needs. Code as compatibility or complexity for institution-related workflow/change process. - Can innovation be changed or adapted to fit the needs of the cancer program or surgeons? - Code answers to Q about ‘can this be adopted in your setting?’   Inclusion Criteria: Include statements regarding the (in)ability to adapt the innovation to their context, e.g., complaints about the rigidity of the protocol. Suggestions for improvement can be captured in this code but should not be included in the rating process, unless it is clear that the participant feels the change is needed but that the program cannot be adapted. However, it may be possible to infer that a large number of suggestions for improvement demonstrates lack of compatibility, see exclusion criteria below.  Exclusion Criteria: Exclude or double code statements that the innovation did or did not need to be adapted to [Compatibility](http://cfirwiki.net/wiki/index.php?title=Compatibility).   - Are you changing the innovation or the inner setting to implement? This will help distinguish between Adaptability and Compatbility. | “Yeah, I think, probably depending on some of those conversations, if there is the ability to be flexible and what we're requiring. So, you know, can a fellow support in this documentation? Can, can we look at, you know, I'm not in the weeds enough with it yet to know but I think we will learn and get feedback on things that might make those physicians lives a little bit easier but still accomplish the standard.”  “And so, I think finding a way to do it that lets people feel like they still have some sort of freedom to do what they wanted to do and still produce the synoptic report is going to be the challenge” |
| 1. Trialability | Definition: The ability to test the innovation on a small scale in the organization, and to be able to reverse course (undo implementation) if warranted.  Inclusion Criteria: Include statements related to whether the site piloted the innovation in the past or has plans to in the future, and comments about whether they believe it is (im)possible to conduct a pilot.   - Include explicit mention of a pilot   Exclusion Criteria: Exclude or double code descriptions of use of results from local or regional pilots to [Evidence Strength & Quality](http://cfirwiki.net/wiki/index.php?title=Evidence_Strength_%26_Quality). | “ …unless there's pushback from other levels, but I think as of now from within the breast team, it should be easily implemented. Well at least trialed. Let's say we do it and, you know, uptake is low or only a small percentage of the notes are, you know, have been revised or have been changed, then it can be bumped up to higher levels because sometimes you know you have to incentivize physicians, whether it's a positive or negative reinforcement, you know.” |
| 1. Complexity | Definition: Perceived **difficulty** of the innovation, reflected by duration, scope, radicalness, disruptiveness, centrality, and intricacy and number of steps required to implement.  Code negative attitudes/perceived difficulty as COMPLEXITY. Code positive attitudes/perceived ease of implementation as COMPATIBILITY.  Inclusion Criteria: Code statements regarding the complexity of the innovation itself.   - Include statements about ‘extra clicks’ to do SOR - Include statements about difficulty of innovation itself - Include statements about change in surgeon workflow - Include statements about SOR not fitting in with current workflow/processes, as it relates to surgeon or institution - Include statements about ‘a lot of time is needed’ - Include statements about ‘large # of the resources to implement.’   Exclusion Criteria: Exclude statements regarding the complexity of implementation and code to the appropriate CFIR code, e.g., difficulties related to space are coded to Available Resources and difficulties related to engaging participants in a new program are coded to [Engaging](http://cfirwiki.net/wiki/index.php?title=Engaging): Innovation Participants.   - Simply mentioning the IT/analyst effort required to implement SOR is coded as Available Resources. | “Well, I mean it's a little, it is some short, yeah, short term pain of setting it up and getting buy-in from everybody…”  “Well, I think that our EHR systems and our ability to do them from a logistics standpoint is not—is not going to fit in immediately with our workflow.”  “I think one of the reasons that we haven't proceeded into the realm of operative reports is that in order to do this at an organization like ours, which is rather large, and because smart forms are not overly laborious to build, but they take a little bit longer than kind of a standard template, we would insist on operational, pardon the pun, agreement on the standard content. We would not be able to maintain a universe where every surgeon has their own version.” |
| 1. Design Quality & Packaging | Definition: Perceived excellence in how the innovation is bundled, presented, and assembled.    Inclusion Criteria: Include statements regarding the quality of the materials and packaging.  (Will likely rarely use this since this would be in relation to actually seeing the implementation materials which the surgeons at this point don’t really have access to)  Exclusion Criteria: Exclude statements regarding the presence or absence of materials and code to [Available Resources](http://cfirwiki.net/wiki/index.php?title=Available_Resources).  Exclude statements regarding the receipt of materials as an engagement strategy and code to [Engaging](http://cfirwiki.net/wiki/index.php?title=Engaging). |  |
| 1. Cost | Definition: Costs of the innovation and costs associated with implementing the innovation including investment, supply, and opportunity costs.  Inclusion Criteria: Include statements related to the cost of the innovation and its implementation.   - Include statements about actual expenses like hiring more people or buying equipment   Exclusion Criteria: Exclude statements related to physical space and time, and code to [Available Resources](http://cfirwiki.net/wiki/index.php?title=Available_Resources). In a research study, exclude statements related to costs of conducting the research components (e.g., funding for research staff, participant incentives).   - Exclude time it takes to develop SOR templates | “ You know, I've only been able to get, like I said, about a third of the surgeons to do direct entry operative notes. And I'll admit part of that is because dictation costs money and direct entry is free and people are people that are good at building personal smart phrases can do it at the samea mount of time. So no, no loss there for the surgeon. It has to be some more of that. It's got to be seen as a win by the surgeons or you're really going to struggle making the conversion.” |
| 1. **Outer Setting** |  |  |
| 1. Needs & Resources of Those Served by the Organization | Definition: The extent to which the needs of those served by the organization [organization here refers to CoC and interaction of the cancer programs with the CoC] as well as barriers and facilitators to meet those needs, are accurately known and prioritized by the organization.    Inclusion Criteria: Include statements demonstrating (lack of) awareness of the needs and resources of those served by the organization. Analysts may be able to infer the level of awareness based on statements about: 1. Perceived need for the innovation based on the needs of those served by the organization and if the innovation will meet those needs; 2. Barriers and facilitators of those served by the organization to participating in the innovation; 3. Participant feedback on the innovation, i.e., satisfaction and success in a program. In addition, include statements that capture whether or not awareness of the needs and resources of those served by the organization influenced the implementation or adaptation of the innovation.   - Include statements about CoC’s lack of awareness of needs/resources from the cancer program.   Exclusion Criteria: Exclude statements that demonstrate a strong need for the innovation and/or that the current situation is untenable and code to [Tension for Change](http://cfirwiki.net/wiki/index.php?title=Tension_for_Change).  Exclude statements related to engagement strategies and outcomes, e.g., how innovation participants became engaged with the innovation, and code to [Engaging](http://cfirwiki.net/wiki/index.php?title=Engaging): Innovation Participants. | “And this might be where the CoC may be overstepped a little bit to assume that everyone has the resources to implement at this level. You know, we can make it happen if there is a true benefit, and but six to nine months of analyst time is pretty significant.” |
| 1. Cosmopolitanism | Definition: The degree to which an organization is networked with other external organizations. [organization here refers to various different cancer programs and the CoC].  Inclusion Criteria: Include descriptions of outside group memberships and networking done outside the organization.   - Include statements about receiving emails or webinars from CoC - Include statements about resources for implementation coming from CoC - Include statements about interacting with other hospitals to share information - Include statements about interacting with Epic to share information - Include statements about knowledge of the extent to which other institutions are implementing the SOR. Double code + TDF Beliefs.   Exclusion Criteria: Exclude statements about general networking, communication, and relationships in the organization, such as descriptions of meetings, email groups, or other methods of keeping people connected and informed, and statements related to team formation, quality, and functioning, and code to [Networks & Communications](http://cfirwiki.net/wiki/index.php?title=Networks_%26_Communications). | “I received the standard CoC emails with any updates and probably at first it was the 2020 phase-in standards. Since then, I have watched the--it wasn't synoptic Op—it was the path synoptic reporting webinar, have been kind of keeping up on all of the updates on whether or not reporting templates would be released from the CoC.” |
| 1. Peer Pressure | Definition: Mimetic or competitive pressure to implement an innovation, typically because most or other key peer or competing organizations [peer organizations = different cancer programs] have already implemented or are in a bid for a competitive edge.  Inclusion Criteria:   - Include statements about perceived pressure or motivation from other entities or organizations in the local geographic area or system to implement the innovation. | “I would say no, because I think for me know, I mean, not to say—it is always good and in general, in my role within the organization, I always appreciate kind of learning from best practices and sharing with other organizations. But I think for us in something like this with synoptic reports in our operating standards, you know, I think we as an organization always hold ourselves to certain standards and we want to be doing the best that we can. And so, I don't think we really change our practices based on what our peers are doing. But really knowing that if this is an expectation of professional organizations, national standards, we will look at guidelines. Excuse me. You know, all of that that we are holding ourselves to those kind of national standards more than what our peers are doing.” |
| 1. External Policy & Incentives | Definition: A broad construct that includes external strategies to spread innovations including policy and regulations (governmental or other central entity - CoC), external mandates, recommendations and guidelines, pay-for-performance, collaboratives, and public or benchmark reporting.  Statements related to the institution’s internal discussion of requirements for CoC accreditation, meeting those requirements, reporting implementation of SOR back to CoC, CoC site visit or survey.  Inclusion Criteria:   - Include descriptions of external performance measures from the system. - Include statements about SOR being a mandate or standard from CoC - Including statements about importance/value/effects of CoC accreditation. Double code + TDF Beliefs. | “Um I, I was planning on doing it anyway, but I mean I think making it a mandate just makes it—forces the institution to do it eventually. Hopefully.”  “Well, ultimately, if it's a CoC standard and we have to comply by those standards, you know, when we get re-accredited, it's going to be, it's going to be looked upon and reflected negatively. Yeah, it would be unfortunate if you know for that demerit that we would not be accredited or we'd be on probation, for example.” |
| 1. **Inner Setting** |  |  |
| 1. Structural Characteristics | Definition: The social architecture, age, maturity, and size of an organization [refers to the cancer program].  Inclusion Criteria:   - Include statements about # of surgeons at the institution - Include statements about academic vs community site - Include statements about structure of committees within the cancer program or institution - Include statements about organizational structure - include descriptions of context of institution within region or nation (institution is in a physician shortage area) - include descriptions about chain of command within institution | “ But I think this is sort of in alignment with what we just talked about, about the benefits of discrete data and the evidence that may be out there. So, I think it's a good thing that the CoC is doing it. It might be more challenging for those nonacademic institutions to implement. Right. They don’t probably have the resources in place. So, but for our institution, I think it's a great move and really in alignment with the benefits.” |
| 1. Networks & Communications | Definition: The nature and quality of webs of social networks, and the nature and quality of formal and informal communications within an organization [refers to the cancer program].  Inclusion Criteria:   - Include statements about general networking, communication, and relationships in the organization, such as descriptions of meetings, email groups, or other methods of keeping people connected and informed, and statements related to team formation, quality, and functioning. - Include statements about internal communications within the cancer program   Exclusion Criteria: Exclude statements related to implementation leaders' and users' access to knowledge and information regarding using the program, i.e., training on the mechanics of the program and code to [Access to Knowledge & Information](http://cfirwiki.net/wiki/index.php?title=Access_to_Knowledge_%26_Information).  Exclude statements related to engagement strategies and outcomes, e.g., how key stakeholders became engaged with the innovation and what their role is in implementation, and code to [Engaging](http://cfirwiki.net/wiki/index.php?title=Engaging): Key Stakeholders.  Exclude descriptions of outside group memberships and networking done outside the organization and code to [Cosmopolitanism](http://cfirwiki.net/wiki/index.php?title=Cosmopolitanism). | “Yes, when the 2020 standards came out, we saw that that was a phase-in standard and began convening meetings to plan for it.” |
| 1. Culture | Definition: Norms, values, and basic assumptions of a given organization  Use CFIR Culture for statements about inner setting (cancer program/institution). Use TDF: Organisational climate/culture for statements about culture of an external group.    Inclusion Criteria:  Include statements about surgeons referencing how they normally dictate their op reports vs using templates  Include statements about individualistic versus cohesive breast group  Include statements related to culture change/remaining the same after implementation of SOR (e.g., stubbornness, changing ways)  Double coding:  Can double code with TDF Breaking Habit  Inclusion criteria, and potential sub-codes, will depend on the framework or definition used for “culture.” For example, if using the [Competing Values Framework](http://www.implementationscience.com/content/2/1/13/abstract) (CVF), you may include four sub-codes related to the four dimensions of the CVF and code statements regarding one or more of the four dimension in an organization.  Exclusion Criteria:   - Exclude statements about culture of an external group. Code to TDF Organizational climate/culture instead. | “ I think it varies depending on general biases about documentation and additional clicks and additional documentation. The younger, more research focused faculty, I would say, not to completely lump people together, but people who understand the value of discrete fields and research and reporting, you know, they're already aware that this is coming or they're already using some sort of a form of this in their outpatient practice, so I think it's going to be an easy pill for some to swallow. For others that are used to their smart phrase narrative reporting, it’s going to be a bit of a change management process.” |
| 1. Implementation Climate | Definition: The absorptive capacity for change, shared receptivity of involved individuals to an innovation, and the extent to which use of that innovation will be rewarded, supported, and expected within their organization.  Inclusion Criteria: Include statements regarding the general level of **receptivity** to implementing the innovation.   - Include statements even if referring to a single individual’s receptivity   Exclusion Criteria: Exclude statements regarding the general level of receptivity that are captured in the sub-codes. | “The younger, more research focused faculty, I would say, not to completely lump people together, but people who understand the value of discrete fields and research and reporting, you know, they're already aware that this is coming or they're already using some sort of a form of this in their outpatient practice, so I think it's going to be an easy pill for some to swallow. For others that are used to their smart phrase narrative reporting, it’s going to be a bit of a change management process.” |
| 1. Tension for Change | Definition: The degree to which stakeholders perceive the current situation as intolerable or needing change.  Inclusion Criteria: Include statements that (do not) demonstrate a strong need for the innovation and/or that the current situation is untenable, e.g., statements that the innovation is absolutely necessary or that the innovation is redundant with other programs. Note: If a participant states that the innovation is redundant with a preferred existing program, (double) code lack of [Relative Advantage](http://cfirwiki.net/wiki/index.php?title=Relative_Advantage), see exclusion criteria below.   - Inclusion should be a strong or explicit statement about the need for the innovation   Exclusion Criteria: Exclude statements regarding specific needs of individuals that demonstrate a need for the innovation, but do not necessarily represent a strong need or an untenable status quo, and code to [Needs and Resources of Those Served by the Organization.](http://cfirwiki.net/wiki/index.php?title=Patient_Needs_%26_Resources)  Exclude statements that demonstrate the innovation is better (or worse) than existing programs and code to [Relative Advantage](http://cfirwiki.net/wiki/index.php?title=Relative_Advantage). | SPEAKER2: it sounds like different groups and different surgeons have different perceptions of the synoptic op report. And you had mentioned earlier that one surgeon thought that the conventional op report is just too complex to be captured by the synoptic format. And so, I'm curious if you could discuss a little bit about the extent that the surgeons feeling the need to use the synoptic op report.  SPEAKER1: Or the resistance you mean?  SPEAKER2: Do they do they feel that they need to use it or are they—?  SPEAKER1: I don't think so. That individual doesn't feel the need to. |
| 1. Compatibility | Definition: The degree of tangible fit between meaning and values attached to the innovation by involved individuals, how those align with individuals’ own norms, values, and perceived risks and needs, and how the innovation fits with existing workflows and systems.  Code negative attitudes/perceived difficulty as COMPLEXITY. Code positive attitudes/perceived ease of implementation as COMPATIBILITY.  Inclusion Criteria: Include statements that demonstrate the level of compatibility the innovation has with organizational values and work processes. Include statements that the innovation did or did not need to be adapted as evidence of compatibility or lack of compatibility.   - Include statements about innovation aligning with institution’s values.   Exclusion Criteria: Exclude or double code statements regarding the priority of the innovation based on compatibility with organizational values to [Relative Priority](http://cfirwiki.net/wiki/index.php?title=Relative_Priority), e.g., if an innovation is not prioritized because it is not compatible with organizational values. | “ I believe it really is the way to track data and get correct accurate data and actually eventually make our EHR more efficient.” |
| 1. Relative Priority | Definition: Individuals’ shared perception of the importance of the implementation within the organization.  Inclusion Criteria: Include statements that reflect the relative priority of the innovation, e.g., statements related to change fatigue in the organization due to implementation of many other programs.   - Competition for analyst time or staff time for implementation   Exclusion Criteria: Exclude or double code statements regarding the priority of the innovation based on compatibility with organizational values to [Compatibility](http://cfirwiki.net/wiki/index.php?title=Compatibility), e.g., if an innovation is not prioritized because it is not compatible with organizational values. | “…a couple of months of time for analysts build and we've got a lot of prioritized projects that are kind of in competition for those analysts’ time. So, I think the perception is just, probably, but it's a really large undertaking and it may need to be prioritized for a later date to allow for that dedicated analyst time.” |
| 1. Organizational Incentives & Rewards | Definition: Extrinsic incentives such as goal-sharing, awards, performance reviews, promotions, and raises in salary, and less tangible incentives such as increased stature or respect.  Inclusion Criteria: Include statements related to whether organizational incentive systems are in place to foster (or hinder) implementation, e.g., rewards or disincentives for staff engaging in the innovation.   - Include incentives, disincentives/punishment, and no incentives. | “ But it will be punitive to the institution, not necessarily to the surgeon. But I think, I don't know, I think it'll be important for those people to know all these benefits that I've described to you about why synoptic reporting is better and what we've really shown.” |
| 1. Goals & Feedback | Definition: The degree to which goals are clearly communicated, acted upon, and fed back to staff, and alignment of that feedback with goals.  Code as Goals & Feedback any time they are talking about compliance of completing breast SOR as a group, and processes for measuring compliance.  Inclusion Criteria: Include statements related to the (lack of) alignment of implementation and innovation goals with larger organizational goals, as well as feedback to staff regarding those goals, e.g., regular audit and feedback showing any gaps between the current organizational status and the goal. Goals and Feedback include organizational processes and supporting structures independent of the implementation process. Evidence of the integration of evaluation components used as part of “Reflecting and Evaluating” into **on-going or sustained** organizational structures and processes may be (double) coded to Goals and Feedback.   - Statements on whether innovation can be adapted at the cancer program can be double coded as Adaptability – innovation characteristics. - Statements about monitoring or reporting surgeon compliance for the purposes of reporting back to CoC should be double coded + External Policy & Incentives   Exclusion Criteria: Exclude statements that refer to the implementation team’s (lack of) assessment of the progress toward and impact of implementation, as well as the interpretation of outcomes related to implementation, and code to [Reflecting & Evaluating](http://cfirwiki.net/wiki/index.php?title=Reflecting_%26_Evaluating). Reflecting and Evaluating is part of the implementation process; it likely ends when implementation activities end. It does not require goals be explicitly articulated; it can focus on descriptions of the current state with real-time judgment, though there may be an implied goal (e.g., we need to implement the innovation) when the implementation team discusses feedback in terms of adjustments needed to complete implementation. | “ We would then get our analysts lined up and we would have a work group set up that would include probably our lead physicians that have been involved to take. So, Dr. [name], Dr. [name], Dr. [name], to be somewhat multidisciplinary, multi-specialty, and then we would draft out the synoptic op reports and then circulate those to the other surgeons on those teams. So really, that's part of kind of more the change management, stakeholder engagement, just making sure that everyone understands there's a requirement coming, they feel like part of the process and like they've provided feedback in the process and then, and then once we get sign off, which may be a process in itself, would kind of wait on the EPIC build.” |
| 1. Learning Climate | Definition: A climate in which: 1. Leaders express their own fallibility and need for team members’ assistance and input; 2. Team members feel that they are essential, valued, and knowledgeable partners in the change process; 3. Individuals feel psychologically safe to try new methods; and 4. There is sufficient time and space for reflective thinking and evaluation.  Inclusion Criteria: Include statements that support (or refute) the degree to which key components of an organization exhibit a “learning climate.” | “ I think it really just—I haven't sat down with all of the clinicians, but I think it just comes down to understanding what the understanding of synoptic reporting is and understanding the point of view from each of the clinicians and addressing the concerns. For example, if someone is concerned that it's not going to adequately reflect the complexity of a procedure, then we should address that. If it's a matter of saying, oh, it's going to create, you know, 15 minutes more time of documentation then we should address that to streamline the process for that person” |
| 1. Readiness for Implementation | Definition: Tangible and immediate indicators of organizational commitment to its decision to implement an innovation.  Inclusion Criteria: Include statements regarding the general level of readiness for implementation.  Exclusion Criteria: Exclude statements regarding the general level of readiness for implementation that are captured in the sub-codes. | “ I think that we wouldn't probably do it without such a guideline. And it's one of the first times that the CoC is really asking us to do something that we're not already doing.” |
| 1. Leadership Engagement | Definition: Commitment, involvement, and accountability of leaders and managers with the implementation of the innovation. **Named formal leaders of organization, is not necessarily championing or ‘leading’ the SOR implementation. E.g. Chief medical officer, department chair.**  C-suite Leadership such as Chief Medical Office, Chief Medical Information Officer, CEO, COO, etc.  Inclusion Criteria: Include statements regarding the level of engagement of organizational leadership.  Exclusion Criteria: Exclude or double code statements regarding leadership engagement to Engaging: [Formally Appointed Internal Implementation Leaders](http://cfirwiki.net/wiki/index.php?title=Formally_Appointed_Internal_Implementation_Leaders) or [Champions](http://cfirwiki.net/wiki/index.php?title=Champions) *if* an organizational leader is also an implementation leader, e.g., if a director of primary care takes the lead in implementing a new treatment guideline. Note that a key characteristic of this Implementation Leader/Champion is that s/he is also an Organizational Leader. | “ Our title chief information officer has sort of said that, you know, he supports exploring the pilot. We, because we have that sort of blessing, we would take it to our prioritization committee which, in oncology, we have our own prioritization committee and from that they basically put any requests and projects into a lineup of priorities” |
| 1. Available Resources | Definition: The level of resources organizational dedicated for implementation and on-going operations including physical space and time.  Inclusion Criteria: Include statements related to the presence or absence of resources specific to the innovation that is being implemented.  Includes mention of resources, **time** of analyst build  Can double code with Cost if referencing monetary aspects (e.g., pay)  Exclusion Criteria: Exclude statements related to training and education and code to [Access to Knowledge & Information](http://cfirwiki.net/wiki/index.php?title=Access_to_Knowledge_%26_Information).   - Exclude statements about personnel needed. Codes this to Stakeholder type codes instead. Include personnel as a resource only when talking about costs associated with staff or time associated with work.   Exclude statements related to the quality of materials and code to [Design Quality & Packaging](http://cfirwiki.net/wiki/index.php?title=Design_Quality_%26_Packaging).  In a research study, exclude statements related to resources needed for conducting the research components (e.g., time to complete research tasks, such as IRB applications, consenting patients). | “But right now, if we were to try to do it ourselves, I think the time and the total FTEs that we have to cover all of our priorities is really the limiting factor.” -001 |
| 1. **Process** |  |  |
| 1. Planning | Definition: The degree to which a scheme or method of behavior and tasks for implementing an innovation are developed in advance, and the quality of those schemes or methods.  Talking about steps, talking about hypothetically what they need to do. = code to PLANNING  Inclusion Criteria: Include evidence of pre-implementation diagnostic assessments and planning, as well as refinements to the plan.  Double coding:   - If specific people are mentioned doing the implementation process, can double code as Champion or Engaging. | “ So, the nice thing is we have our survey coming up in 2022 and it's not required that we have it implemented, obviously, we just have to have a plan. And so, I think we've got some time for that change management to happen and look forward to hearing more about best practices across the country in terms of how that is being done in an integrated way to not add more administrative” |
| 1. Engaging | Definition: Attracting and involving appropriate individuals in the implementation and use of the innovation through a combined strategy of social marketing, education, role modeling, training, and other similar activities.  If just mentioning people, just include people codes. If process of involving, then double code it as Engaging.  Inclusion Criteria: Include statements related to engagement strategies and outcomes, i.e., if and how staff and innovation participants became engaged with the innovation and what their role is in implementation. Note: Although both strategies and outcomes are coded here, the outcome of engagement efforts determines the rating, i.e., if there are repeated attempts to engage staff that are unsuccessful, or if a role is vacant, the construct receives a negative rating. In addition, you may also want to code the "quality" of staff - their capabilities, motivation, and skills, i.e., how good they are at their job, and this data affects the rating as well.  Exclusion Criteria: Exclude statements related to specific sub constructs, e.g., [Champions](http://cfirwiki.net/wiki/index.php?title=Champions) or [Opinion Leaders](http://cfirwiki.net/wiki/index.php?title=Opinion_Leaders).  Exclude or double code statements related to who participated in the decision process to implement the innovation to [Innovation Source](http://cfirwiki.net/wiki/index.php?title=Intervention_Source), as an indicator of internal or external innovation source. | “ And to be quite honest, we haven't really started to engage those people yet. We've really focused on the people that are going to help us set us up for success. So, for each disease team, we have kind of some champions and super users that we've identified to really understand how this can be best integrated into current workflows and practices.” |
| 1. Opinion Leaders | Definition: Individuals in an organization that have formal or informal influence on the attitudes and beliefs of their colleagues with respect to implementing the innovation  Inclusion Criteria: Include statements related to engagement strategies and outcomes, e.g., how the opinion leader became engaged with the innovation and what their role is in implementation. Note: Although both strategies and outcomes are coded here, the outcome of efforts to engage staff determines the rating, i.e., if there are repeated attempts to engage an opinion leader that are unsuccessful, or if the opinion leader leaves the organization and this role is vacant, the construct receives a negative rating. In addition, you may also want to code the "quality" of the opinion leader here - their capabilities, motivation, and skills, i.e., how good they are at their job, and this data affects the rating as well.  Double coding:   - Always double code with Champion | “I would be the administrative lead for implementing. The physician lead will be our cancer liaison physician, Dr. [name], and Dr. [name] with the colorectal team. Yeah, I typically do more of the project work and they're going to do more of the physician change management—really having those one-on-one conversation so that it's not just an email like this is required do this. It's not going to be well received. So, so it definitely benefits our team to have that physician and administrative dyad.” |
| 1. Formally Appointed Internal Implementation Leaders | Definition: Individuals from within the organization who have been formally appointed with responsibility for implementing an innovation as coordinator, project manager, team leader, or other similar role, only the following: **cancer liaison physician, breast section lead, accreditation manager, IT analyst lead.**  Inclusion Criteria: Include statements related to engagement strategies and outcomes, e.g., how the formally appointed internal implementation leader became engaged with the innovation and what their role is in implementation. Note: Although both strategies and outcomes are coded here, the outcome of efforts to engage staff determines the rating, i.e., if there are repeated attempts to engage an implementation leader that are unsuccessful, or if the implementation leader leaves the organization and this role is vacant, the construct receives a negative rating. In addition, you may also want to code the "quality" of the implementation leader here - their capabilities, motivation, and skills, i.e., how good they are at their job, and this data affects the rating as well.  Exclusion Criteria: Exclude or double code statements regarding leadership engagement to [Leadership Engagement](http://cfirwiki.net/wiki/index.php?title=Leadership_Engagement) *if* an implementation leader is also an organizational leader, e.g., if a director of primary care takes the lead in implementing a new treatment guideline. | “ I think that from a leadership standpoint, I've noticed that things happen better when it's kind of a health systems mandate or if it's supported by the health system. So, if the, if the health system helps in the implementation process and provides resources like IT support or, you know, at least a temporary consultant, I think that would make the transition easier.” |
| 1. Champions | Definition: “Individuals who dedicate themselves to supporting, marketing, and ‘driving through’ an [implementation]”, overcoming indifference or resistance that the innovation may provoke in an organization. **(for example, cancer committee members, accreditation administrator) Can be double coded with Opinion Leaders.**  Inclusion Criteria: Include statements related to engagement strategies and outcomes, e.g., how the champion became engaged with the innovation and what their role is in implementation. Note: Although both strategies and outcomes are coded here, the outcome of efforts to engage staff determines the rating, i.e., if there are repeated attempts to engage a champion that are unsuccessful, or if the champion leaves the organization and this role is vacant, the construct receives a negative rating.  Exclusion Criteria: Exclude or double code statements regarding leadership engagement to [Leadership Engagement](http://cfirwiki.net/wiki/index.php?title=Leadership_Engagement) *if* a champion is also an organizational leader, e.g., if a director of primary care takes the lead in implementing a new treatment guideline. | “ I think I was sort of a natural person to do it, since I'm on the implementation committee through ACS and I've been working with the with the API to develop the—with the group that's making the API to develop that. So, I think that, and I already have physician builder. So, I, for a lot of reasons, it was just natural.” |
| 1. External Change Agents | Definition: Individuals who are affiliated with an outside entity who formally influence or facilitate innovation decisions in a desirable direction.  Inclusion Criteria: Include statements related to engagement strategies and outcomes, e.g., how the external change agent (entities outside the organization that facilitate change) became engaged with the innovation and what their role is in implementation, e.g., how they supported implementation efforts. Note: Although both strategies and outcomes are coded here, the outcome of efforts to engage staff determines the rating, i.e., if there are repeated attempts to engage an external change agent that are unsuccessful, or if the external change agent leaves their organization and this role is vacant, the construct receives a negative rating. In addition, you may also want to code the "quality" of the external change agent here - their capabilities, motivation, and skills, i.e., how good they are at their job, and this data affects the rating as well.   - Include statements about external EPIC or electronic medical record consultant   Exclusion Criteria: Note: It is important to clearly define what roles are external and internal to the organization. Exclude statements regarding facilitating activities, such as training in the mechanics of the program, and code to [Access to Knowledge & Information](http://cfirwiki.net/wiki/index.php?title=Access_to_Knowledge_%26_Information) *if* the change agent is considered internal to the study, e.g., a staff member at the national office. If the study considers this staff member internal to the organization, it should be coded to [Access to Knowledge & Information](http://cfirwiki.net/wiki/index.php?title=Access_to_Knowledge_%26_Information), even though their support may overlap with what would be expected from an External Change Agent. | “ in terms of, you know, why wouldn't they get to it sooner? Or why haven't they got done yet? It's the same thing that's everywhere in I.T. It's that demand far exceeds supply in terms of resources, whether it's personnel or time. Epic has a lot of things that they want to develop for customers, both in terms of end user usability and functionality, so on. They're are also by virtue of being one of the 800 pound gorilla space, they're obligated to respond to a continual stream of new regulatory requirements.” |
| 1. Key Stakeholders | Definition: Individuals from within the organization that are directly impacted by the innovation.   - Key stakeholder = larger group that include people working on SOR implementation who may not be titled leaders.   Inclusion Criteria: Include statements related to engagement strategies and outcomes, e.g., how key stakeholders became engaged with the innovation and what their role is in implementation. Note: Although both strategies and outcomes are coded here, the outcome of efforts to engage staff determines the rating, i.e., if there are repeated attempts to engage key stakeholders that are unsuccessful, the construct receives a negative rating.  Exclusion Criteria: Exclude statements related to implementation leaders' and users' access to knowledge and information regarding using the program, i.e., training on the mechanics of the program, and code to [Access to Knowledge & Information](http://cfirwiki.net/wiki/index.php?title=Access_to_Knowledge_%26_Information).  Exclude statements about general networking, communication, and relationships in the organization, such as descriptions of meetings, email groups, or other methods of keeping people connected and informed, and statements related to team formation, quality, and functioning, and code to [Networks & Communications](http://cfirwiki.net/wiki/index.php?title=Networks_%26_Communications). | “ I think most, I might have a biased view because most of the, you know, I work with a lot of committee members, but most of the people on the committee feel that the Commission on Cancer is doing things that are very valuable or invaluable, that's I guess it's the same thing, invaluable to the standardization of cancer care. And we're trying as much as possible to adhere to those standards or at least or be examples or leaders. But overall positive.” |
| 1. Innovation Participants | Definition: Individuals served by the organization that participate in the innovation   - Breast surgeons only   Inclusion Criteria: Include statements related to engagement strategies and outcomes, e.g., how innovation participants became engaged with the innovation. Note: Although both strategies and outcomes are coded here, the outcome of efforts to engage participants determines the rating, i.e., if there are repeated attempts to engage participants that are unsuccessful, the construct receives a negative rating.  Exclusion Criteria: Exclude statements demonstrating (lack of) awareness of the needs and resources of those served by the organization and whether or not that awareness influenced the implementation or adaptation of the innovation and code to [Needs & Resources of Those Served by the Organization](http://cfirwiki.net/wiki/index.php?title=Patient_Needs_%26_Resources). | “ Well, I think that our EHR systems and our ability to do them from a logistics standpoint is not—is not going to fit in immediately with our workflow. And so changing habits, especially, I think for surgeons is very difficult. For anybody it's difficult, but I don't know, I think surgeons are especially stubborn.” |
| 1. Executing | Definition: Carrying out or accomplishing the implementation according to plan.  Inclusion Criteria: Include statements that demonstrate how implementation occurred with respect to the implementation plan. Note: Executing is coded very infrequently due to a lack of planning. However, some studies have used fidelity measures to assess executing, as an indication of the degree to which implementation was accomplished according to plan. |  |
| 1. Reflecting & Evaluating | Definition: Quantitative and qualitative feedback about the progress and quality of implementation accompanied with regular personal and team debriefing about progress and experience.  Inclusion Criteria: Include statements that refer to the implementation team’s (lack of) assessment of the progress toward and impact of implementation, as well as the interpretation of outcomes related to implementation. Reflecting and Evaluating is part of the implementation process; it likely ends when implementation activities end. It does not require goals be explicitly articulated; it can focus on descriptions of the current state with real-time judgment, though there may be an implied goal (e.g., we need to implement the innovation) when the implementation team discusses feedback in terms of adjustments needed to complete implementation.  Exclusion Criteria: Exclude statements related to the (lack of) alignment of implementation and innovation goals with larger organizational goals, as well as feedback to staff regarding those goals, e.g., regular audit and feedback showing any gaps between the current organizational status and the goal, and code to [Goals & Feedback](http://cfirwiki.net/wiki/index.php?title=Goals_%26_Feedback). Goals and Feedback include organizational processes and supporting structures independent of the implementation process. Evidence of the integration of evaluation components used as part of “Reflecting and Evaluating” into **on-going or sustained** organizational structures and processes may be (double) coded to Goals and Feedback.   - Statements on compliance with SOR use should be Goals & Feedback.   Exclude statements that capture reflecting and evaluating that participants may do during the interview, for example, related to the success of the implementation, and code to [Knowledge & Beliefs about the Innovation](http://cfirwiki.net/wiki/index.php?title=Knowledge_%26_Beliefs_about_the_Intervention). |  |
| **Additional Codes (not CFIR or TDF):** |  |  |
| Uncertainty | Statements about not knowing or questions unanswered. Double code with an existing node such as “Knowledge & Beliefs about the Innovation”, “Complexity”, “Availabe Resources”, etc  *always double code with CFIR or TDF code | “ No, I don't know that I have. If I have, I haven't read it as such. I would say in general, in my role, I understand the value of discrete documentation and all of the downstream effects that make that beneficial. But in terms of clinical care, I can only assume. I can't say that I've read, you know, because I don't know that it's implemented across the board to—to have evidence. But if there's evidence, I'd be happy to review it and read it.” |
| Non-breast SOR | Include statements about non-breast SORs.  Always double code with CFIR or TDF code as appropriate. | “ Well, when I went through for our accreditation for NAPRC, and it took us, probably it took us, probably two years to reliably get people doing their operative notes and what it took actually was, my emails to people sometimes reminded them to do it, and they would sometimes do it, but it really took me every single week at our conference highlighting when we reviewed their post-operative cases, when we reviewed their post-operative pathology, saying we need your synoptic note, you did not do your synoptic note, and doing that in front of that group. And I think people that didn't like that, and they finally started doing them and I could stop doing it after a while, just because people were doing them. But I think that actually, unfortunately, is really what it took.” |

| **TDF: Knowledge** | **Definition** | **Examples** |
| --- | --- | --- |
| Knowledge | An awareness of the existence of something. [Stakeholder knows what SOR is, doesn’t necessarily need to know how to do it. Exclude statements about attitude regarding the SOR]  Inclusion Criteria:   - Include statements about awareness of SOR or CoC standard - Include statements about technical elements included in the breast SOR (i.e., sentinel lymph node biopsy, axillary dissection, tracers, other “synoptic lanagauge”) - Descriptions of synoptic format in general (discrete fields, checklist, etc.) - Descriptions of timeline for implementing SOR set forth by CoC à double code with External Policy - Descriptions of requirements that institutions must fulfill to maintain accreditation à double code with External Policy   Exclude:   - Statements about implementation and planning - Statements about advantages/disadvantages. Code to Relative Advantage. - Statements about attitude regarding SOR | “My understanding is that there are defined fields for four different procedure areas that need to be documented as part of the surgical reports, and they are discrete fields that can then be reported on.” |
| **TDF: Skills** |  |  |
| Skills | An ability or proficiency acquired through training and/or practice.  Inclusion Criteria:   - Include statements about training - Include statements about previous experience - Include statements about other people, not just speaker themselves | “…and it's probably influenced mostly by my training in biomedical informatics, my desire to research and improve the EHR data tracking.” |
| **TDF: Beliefs about capabilities** |  |  |
| Self-efficacy | An individual's capacity to act effectively to bring about desired results, as perceived by the individual  Inclusion:   - Include statements only about speaker’s perception of their own capacity to bring about desired results - Include statements about attitudes or perceptions about how easily the speaker can implement SOR (“easy” for someone to build and learn themselves).   Exlcusion:   - Exclude statements about other people, unless explicity says other person is capable. | “I don't think it has that much of an influence, I mean, like I said, it's pretty easy for me to implement. And so, I was, once I have the op report I was planning on implementing in any way. So, I don't think it really influences knowing that it's mandated now.” |
| **TDF: Beliefs about consequences** |  |  |
| Beliefs | The thing believed; the proposition or set of propositions held true.  For statements not directly related to breast SOR or implementation of breast-SOR at the speaker’s instiutiton.  Inclusion:  Include statements such as beliefs about the CoC.  Include statements about how people at their institution perceive CoC or others’ opinions about the CoC  Include statements about beliefs about external organizations like other accrediting bodies or Epic  Include statements about other standards required for accredidations (not other SORs)  Double coding:  If the comment includes implication of how a benefit from another SOR will apply to breast SOR then apply appropriate CFIR/Relative Advantage code + ‘Non-breast SOR’  Exclusion:   - Excluded beliefs about advantages and disadvantages of CoC breast SOR   Exclude statements about other SORs. Code to ‘Non-breast SOR’ instead. | “I think for academic institutions, oftentimes accreditations are generally just sort of, you know, administrative documenting what you're already doing. But I think this is sort of in alignment with what we just talked about, about the benefits of discrete data and the evidence that may be out there. So, I think it's a good thing that the CoC is doing it. It might be more challenging for those nonacademic institutions to implement. Right. They don’t probably have the resources in place. So, but for our institution, I think it's a great move and really in alignment with the benefits.” |
| **TDF: Motivation and goals** |  |  |
| Goal priority | Order of importance or urgency of **external groups** toward goal which one is striving.  Inclusion:   - Include statements about **external groups** involvement with prioritizing the SOR implementation and their timeframe to complete this   Exclusion:   - Exclude statements about the institution itself prioritizing the SOR. Code to CFIR Relative Priority | “in terms of, you know, why wouldn't they get to it sooner? Or why haven't they got done yet? It's the same thing that's everywhere in I.T. It's that demand far exceeds supply in terms of resources, whether it's personnel or time. Epic has a lot of things that they want to develop for customers, both in terms of end user usability and functionality, so on.” |
| **TDF: Memory, attention, and decision processes** |  |  |
| Memory | The ability to retain information or a representation of a past experience, based on the mental processes of learning or encoding retention across some interval of time, and retrieval or reactivation of the memory; specific information of a specific past  Inclusion:   - Include statements about surgeons remembering or forgetting to use SOR | “I think it's possible that people might click that something had been visualized or protected or that maybe they actually hadn't. But all along, our, part of our at least part of my thought process in this had been that even if occasionally they click that they did identify the long thoracic nerve well and protect it when in fact they didn't do that, it reminds them every time they have to click that, that that's part of what they're supposed to be doing. So, in my mind, that would make it more likely that the next case, they'll remember to look against the chest wall and identify the dirty little thing and protect it. And so, I think it's possible that operative reports might include things that are maybe not exactly what a camera over the operative field would have identified, but it still would be an educational thing that repeatedly, every time you report your operation, you're having to click about this long thoracic nerve and whether you identified it, protected it or not. That's got to have some educational benefit. So that's the long answer to I don't think that there's a downside as long as it doesn't take longer to do it than what the surgeons are currently doing.” |
| **TDF: Environmental context and resources** |  |  |
| Environmental stressors | External factors (external to cancer program) in the environment that cause stress. **Include statements that identify what the stressor is**.  Inclusion:   - Include statements about factors external to cancer program/institution such as COVID - **Include statements that identify what the stressor is**. For example, if they talk about COVID causing limited resources, then use TDF Environmental Stressors, Available Resources, and Relative Priority.   Exclusion: exclude statements about factors internal to the cancer program/institution. Code that to CFIR inner setting.   - Exclude statements if talking generally (without explicitly identifying what the environmental stressor is) Ex: “But right now, if we were to try to do it ourselves, I think the time and the total FTEs that we have to cover all of our priorities is really the limiting factor.” -001 code to Available Resources, Relative Priority ONLY. | “Short term, yes. Just physician satisfaction during COVID is low. Documentation burden is at an all-time high. So, the timing is a little bit unfortunate, but I think if we can really kind of make the case for a value add for the patient and for the physician, we can get past that and the long-term benefit will be worth it.” |
| **TDF: Social Influences** |  |  |
| Organizational climate/culture | A distinctive pattern of thought and behavior shared by members of the same organization and reflected in their language, values, attitudes, beliefs and customs.  Inclusion:   - Include statements talking about culture of external groups (e.g., CoC at national level, Society of Surgical Oncology SSO, ASBrS)   Exclusion:   - Exclude statements talking about culture of cancer program/institution. Code to CFIR Culture instead. | “I drank the Kool-Aid” when referencing how the CoC influenced their opinion of the SOR |
| **TDF: Nature of the behavior** |  |  |
| Breaking habit | To discontinue a behavior or sequence of behaviors that is automatically activated by relevant situational cues  Inclusion:   - Include statements about how surgeons are stubborn in their ways, difficult to change habit - Include statements related to any habit surrounding documentation, surgery itself, workflow, etc | “She’s pretty set in her ways. And yeah, she does things the way she does them. And she's not gonna change—I mean I sent her an email that we're doing this study just and I sent it to both her and my other partner and she like didn't even respond. Um you'd have to be very punitive to get her to do it.” |
